# A nomogram for predicting mild cognitive impairment in older adults with hypertension

**DOI:** 10.1186/s12883-023-03408-y

**Published:** 2023-10-09

**Authors:** Lu Jingyu, Ding Wen, Zhang Liping, Liu Xiaoling

**Affiliations:** 1 Nursing Department, General Hospital of Ningxia Medical University, 804 Shengli Street, Xingqing District, Yinchuan, China; 2 Department of Cardiovascular Medicine, General Hospital of Ningxia Medical University, Yinchuan, China; 3 Neurology Department, General Hospital of Ningxia Medical University, Yinchuan, China

**Keywords:** Older adults, Hypertension, Mild cognitive impairments, Nomogram, Predictive model

## Abstract

**Background:**

Hyper- and hypotension increase the risk of cognitive dysfunction. As effective control of blood pressure can reduce the risk of mild cognitive impairment (MCI), early risk assessment is necessary to identify MCI in senile hypertension as soon as possible and reduce the risk of developing dementia. No perfect risk-prediction model or nomogram has been developed to evaluate the risk of MCI in older adults with hypertension. We aimed to develop a nomogram model for predicting MCI in older patients with hypertension.

**Methods:**

We selected 345 older patients with hypertension in Xixiangtang District, Nanning City, as the modeling group and divided into the MCI (*n* = 197) and non-MCI groups (*n* = 148). Comparing the general conditions, lifestyle, disease factors, psychosocial and other indicators. Logistic regression was used to analyze risk factors for MCI in older hypertensive patients, and R Programming Language was used to draw the nomogram. We selected 146 older patients with hypertension in Qingxiu District, Nanning City, as the verification group. The effectiveness and discrimination ability of the nomogram was evaluated through internal and external verification.

**Results:**

Multivariate logistic regression analysis identified 11 factors, including hypertension grade, education level, complicated diabetes, hypertension years, stress history, smoking, physical exercise, reading, social support, sleep disorders, and medication compliance, as risk factors for MCI in older patients with hypertension. To develop a nomogram model, the validity of the prediction model was evaluated by fitting the curve, which revealed a good fit for both the modeling (*P* = 0.98) and verification groups (*P* = 0.96). The discrimination of the nomogram model was evaluated in the modeling group using a receiver operating characteristic curve. The area under the curve was 0.795, and the Hosmer–Lemeshow test yielded *P* = 0.703. In the validation group, the area under the curve was 0.765, and the Hosmer–Lemeshow test yielded *P* = 0.234.

**Conclusions:**

We developed a nomogram to help clinicians identify high-risk groups for MCI among older patients with hypertension. This model demonstrated good discrimination and validity, providing a scientific basis for community medical staff to evaluate and identify the risk of MCI in these patients at an early stage.

## Background

Mild cognitive impairment (MCI) is a transitional state between normal aging and dementia. It is characterized by various degrees of age-related memory loss or cognitive impairment, which do not affect daily life. MCI is considered an early warning for dementia development [1]. The risk of developing dementia in patients with MCI is three to five times that in individuals with normal cognitive function, and approximately 6.53% of patients with MCI develop dementia every year [2]. Nevertheless, impaired cognitive function in patients with MCI is reversible, and it is the best stage for preventive intervention for dementia. If reasonable intervention is performed, dementia can be delayed or avoided.

In people aged > 70 years, the incidence of MCI is nearly 20%, increasing with age. Moreover, many studies [3, 4] have shown that hypertension increases the risk of dementia and cognitive impairment. According to the American Heart Association [5], middle-aged hypertensive patients are likely to have cognitive dysfunction in their later years, which brings great burden to their families and society. The 2018 Guidelines for Diagnosis and Treatment of Dementia and Cognitive Impairment in China [6] also points out that hypertension is a preventable risk factor for cognitive dysfunction and that the incidence and severity of cognitive dysfunction are positively related to the course of hypertension. Some scholars' longitudinal studies show that the elderly with higher systolic or diastolic blood pressure have higher proportion of cognitive impairment in the future. It may be that high blood pressure will destroy the cerebrovascular structure, accelerate atherosclerosis, damage the important cerebrovascular regulation mechanism, increase the susceptibility to cerebral ischemia injury, destroy the white matter structure of the brain, and interrupt the connection between functional areas of the brain [7]. The China older hypertension management guide 2019 [8] also points out that early screening of cognitive function is recommended for elderly patients with hypertension, so as to assess the risk and severity of cognitive dysfunction and intervene in advance. Therefore, early risk assessment to identify MCI in older adults with hypertension as soon as possible is vital for reducing the risk of dementia.

A nomogram is a combination of several specific predictors that can estimate the absolute probability and risk of an individual’s outcome and is often used to evaluate and predict the risk of disease occurrence and prognosis. Its advantage lies in presenting complex regression equations in graphs and scoring tables, allowing more convenient interpretation and evaluation of results by doctors and nurses [9]. At present, nomograms have been applied in many research fields, such as tumors, chronic diseases, and gynecology. In terms of cognitive function, many researchers have established nomograms for the risk of cognitive dysfunction in patients with stroke, cerebral small vessel disease, and supratentorial spontaneous intracranial hematoma [10–12]. However, no perfect risk-prediction model or nomogram has been developed to evaluate the risk of MCI in older adults with hypertension.

Therefore, given this background, the present study sought to analyze clinical data related to cognitive function in older patients with hypertension and to establish a nomogram as a risk-prediction model to provide a basis for clinical medical staff to evaluate and intervene in terms of the risk of MCI in older patients with hypertension.

## Methods

### Study design and patients

This was a retrospective, observational study, convenient sampling was used in the study. Older adults with hypertension living in a community in the Xixiangtang District of Nanning, China, between April and July 2020 were selected as the research subjects for the modeling group. Older adults with hypertension living in a community in the Qingxiu District, Nanning, China, between August and October 2020, were selected as the research subjects for the verification group. The inclusion criteria were a diagnosis of hypertension [13], age ≥ 65 years, ability to read and write independently or complete the questionnaire with the help of investigators, and providing informed consent to participate in this study. The exclusion criteria were serious mental disorders or physical illness; secondary hypertension; previously diagnosed neurological diseases, such as Alzheimer's disease, mixed dementia, Parkinson's disease, and multiple sclerosis; and the use of sedative or psychotropic drugs in the last month.

The following was according to the sample size estimation method of a multivariate logistic regression model [14]: *n* = independent variable × (5–10) ÷ prevalence rate. Based on a literature review, 28 variables were identified, and the study required 5–10 patients for each independent variable. According to previous reports [9–17], the incidence of MCI in patients with hypertension in China is approximately 52%. Considering 20% invalid samples, the sample size required in this study was at least 28 × 5 ÷ 52% ÷ (1–0.2) = 337. The final actual sample size of the modeling group in this study was 345 cases.

### Definitions

Hypertension was defined based on the diagnostic criteria of the National Institute for Health and Care Excellence’s new hypertension guidelines [13] as systolic blood pressure ≥ 140 mmHg (1 mmHg = 0.133 kPa) and/or diastolic blood pressure ≥ 90 mmHg. Grade 1 hypertension was defined as follows: systolic blood pressure = 140–159 mmHg and/or diastolic blood pressure = 90–99 mmHg. Grade 2 hypertension was defined as follows: systolic blood pressure = 160–179 mmHg and/or diastolic blood pressure = 100–109 mmHg.

MCI was defined by the diagnostic criteria of Petersen et al. [15] and the “Diagnosis and Treatment of Dementia and Cognitive Impairment in China in 2018 [6].” The chief complaint is memory loss lasting 3 months or more, the total Montreal Cognitive Assessment [MoCA] scale score of < 26, no dementia diagnosis (excluded by the Mini-Mental State Examination [MMSE] score), normal or slightly impaired abilities of daily living (Activities of Daily Living [ADL] < 22 points), and the absence of special reasons that may cause cognitive decline.

The members of the research team include a deputy chief physician of neurology, a nurse and two graduate students. After screening and consultation through questionnaires, they jointly diagnosed whether the patient had mild cognitive impairment according to the diagnostic criteria.

### Data acquisition

The general information questionnaire included demographic information, disease-related information, lifestyle-related information, and exercise habits. Among these, smoking was defined as either having a smoking history or smoking at present. Two response options, “occasionally” and “often,” were provided for exercise/reading/housework frequencies. Occasionally was described as performing the activity at least once a month and often as performing the activity at least once a week. The term complicated with diabetes was defined as the patient having been diagnosed with diabetes by a doctor. Table 1 includes further details. The MoCA was used to evaluate the cognitive status of older adults with hypertension. Its Cronbach’s α coefficient was 0.818. The MMSE was used to exclude dementia patients, and its Cronbach’s α coefficient was 0.890 [16]. The ADL scale was used to evaluate the daily living abilities of the participants and had a Cronbach’s α coefficient of 0.75 [17]. The Morisky Medication Compliance scale was used to assess medication compliance. Its Cronbach’s α coefficient was 0.83. The Pittsburgh Sleep Quality Index (PSQI) was used to evaluate sleep status. The PSQI was compiled by Buysse et al. [18] in 1989 and reflects the sleep situation of patients in the past month. The Hospital Anxiety and Depression Scale was used to evaluate anxiety and depression. The total Cronbach’s α coefficient of the scale was 0.879 [19]. The Social Support Rate Scale was used to assess social support, and Cronbach’s α for each dimension was 0.89–0.94 [20].

**Table 1:** Univariate analysis of factors associated with mild cognitive impairment in older adults with hypertension

Characteristics	Non-MCI (*n* = 148)	MCI (*n* = 197)	Statistic	*P-*value
Sex			χ^2^ = 3.194	0.074
Male	82(55.4%)	90(45.7%)		
Female	66(44.6%)	107(54.3%)		
Age			χ^2^ = 27.585	< 0.001
65–69	72(48.6%)	63(32.0%)		
70–74	32(21.6%)	57(28.8%)		
75–79	29(19.6%)	20(10.2%)		
80–84	8(5.4%)	35(17.8%)		
85–89	7(4.8%)	22(11.2%)		
BMI			χ^2^ = 3.795	0.285
< 18.5	4(2.7%)	8(4.1%)		
18.5–23.9	61(41.2%)	90(45.7%)		
24–27.9	58(39.2%)	79(40.1%)		
≥ 28	25(16.9%)	20(10.2%)		
Education Level			χ^2^ = 39.794	< 0.001
Primary school and below	4(2.7%)	45(22.8%)		
Junior school	51(34.5%)	82(41.6%)		
Technical Secondary school/High school	66(44.6%)	44(22.3%)		
College or above	27(18.2%)	26(13.3%)		
Marital Status			χ^2^ = 4.146	0.042
Have a Spouse	135(91.2%)	165(83.8%)		
No Spouse	13(8.8%)	32(16.2%)		
Living Condition			χ^2^ = 6.375	0.012
Non-solitary	142(95.9%)	174(88.3%)		
Solitary	6(4.1%)	23(11.7%)		
Hypertension Course (year)			χ^2^ = 41.069	< 0.001
< 5	56(37.8%)	27(13.7%)		
5 ~ 10	60(40.6%)	68(34.5%)		
> 10	32(21.6%)	102(51.8%)		
Hypertension Classification			χ^2^ = 11.564	0.001
Grade 1	109(73.6%)	110(55.8%)		
Grade 2	39(26.4%)	87(44.2%)		
Number of drugs taken			χ^2^ = 0.534	0.766
1	64(43.2%)	82(41.6%)		
2	73(49.3%)	96(48.7%)		
3	11(7.4%)	19(.6%)		
Medication Compliance			χ^2^ = 31.474	0.001
Low	65(43.9%)	143(72.6%)		
Middle	54(36.5%)	42(21.3%)		
High	29(19.6%)	12(6.1%)		
Stress History in the past year			χ^2^ = 14.024	< 0.001
No	126(85.1%)	133(67.5%)		
Yes	22(14.9%)	64(32.5%)		
History of falls in the past year			χ^2^ = 3.393	0.065
No	137(92.6%)	170(86.3%)		
Yes	11(7.4%)	27(13.7%)		
Complicated with diabetes			χ^2^ = 16.663	< 0.001
No	114(77.0%)	110(55.8%)		
Yes	34(23.0%)	87(44.2%)		
Smoking			χ^2^ = 13.525	< 0.001
No	107(72.3%)	104(52.8%)		
Yes	41(27.7%)	93(47.2%)		
Physical Exercise			χ^2^ = 11.371	0.003
Never	13(8.8%)	38(19.3%)		
Occasionally	41(27.7%)	66(33.5%)		
Often	94(63.5%)	93(47.2%)		
Reading			χ^2^ = 12.908	0.002
Never	108 (73.0%)	173 (87.8%)		
Occasionally	30 (20.3%)	20 (10.2%)		
Often	10 (6.7%)	4 (2.0%)		
Do housework			χ^2^ = 2.200	0.333
Never	3 (2%)	8(4.1%)		
Occasionally	75 (50.7%)	87(44.2%)		
Often	70 (47.3&)	102(51.8%)		
Sleep disorder			χ^2^ = 18.199	< 0.001
No	86(58.1%)	69(35%)		
Yes	62(41.9%)	128(65%)		
Anxiety			χ^2^ = 11.136	0.001
No	124(83.8%)	134(68%)		
Yes	24(16.2%)	63(32%)		
Depression			χ^2^ = 12.931	< 0.001
No	128(86.5%)	138(70.1%)		
Yes	20(13.5%)	59(29.9%)		
Social Support	37.67 ± 6.85	33.59 ± 7.30	t = 5.281	< 0.001

*MCI* mild cognitive impairment

All the researchers have been trained by neurologists to make clear the use method and the meaning of the questionnaire. The survey was conducted face-to-face on the spot. First, the purpose, content and filling method of the survey were introduced to the subjects with unified instructions, and the questionnaires were distributed after obtaining their informed consent. In principle, the contents of the questionnaire should be filled in by the subjects. However, for those subjects who cannot fill in the questionnaire by themselves due to poor eyesight and attention, the researchers should help them fill in the questionnaire after asking the accurate answers of each item, and pay attention to avoiding subjective induced answers. Questionnaires will be distributed on the spot, and guidance will be given to fill in, collect on the spot and check whether there are any mistakes or omissions.

### Statistical analysis

EpiData 3.1 (https://www.epidata/dk) was used to input data, and SPSS (version 23.0; IBM SPSS Inc., Armonk, NY, USA) was used to analyze the data.

1. Univariate analysis: The measurement data that had a normal distribution and homogeneity of variance were compared between the mild cognitive impairment and non-mild cognitive impairment groups in the modeling group by one-way analysis of variance or two independent-sample t-tests. The Mann–Whitney U test was used to compare the measurement data that did not obey normal distribution or have homogeneity of variance. The chi-square test was used to compare count data between groups.
2. Analysis of risk factors of MCI: We include the meaningful factors in univariate analysis, and used multivariate logistic stepwise regression. Finally, the risk factors of MCI were determined.
3. Construction of nomogram model: Introduce the screened independent risk factors into R software (https://www.r-project.org), draw the nomogram.
4. Verification of nomogram model: The verification of the model includes internal and external verification. It is necessary to verify its discriminant validity (discrimination) and coincidence degree (calibration) respectively. A receiver operating characteristic (ROC) curve and fitting curve was drawn to evaluate the discrimination ability and effectiveness of the model. All the data was verified by a statistician.

## Results

### Clinical characteristics

Among the 345 patients in the modeling group, there were 172 males (49.9%) and 173 females (51.1%). Their age was in the range of 65–88 years (mean [standard deviation]: 73.61 [7.02]) years. Eighty-three cases (24.1%) had a hypertension course < 5 years, 128 cases (37.1%) had a hypertension course of 5–10 years, and 134 cases (38.8%) had a hypertension course of > 10 years. Two-hundred-and-nineteen cases (63.5%) were of grade 1 hypertension, and 126 (36.5%) were of grade 2 hypertension. The incidence of MCI was 57.1% (197/345). Among the 146 patients in the validation group, 78 were male (53.4%) and 68 were female (46.6%). Their age was in the range of 65–87 years (73.20 [6.74]) years, and the incidence of MCI was 55.5% (81/146).

### Univariate and multivariate analyses

According to the diagnostic criteria, the 345 older patients with hypertension in the modeling group were divided into an MCI group (*n* = 197) and a non-MCI group (*n* = 148). The incidence of MCI in the modeling group was 57.1%. The results showed that age, education level, marital status, living conditions, hypertension course, hypertension classification, medication compliance, stress history in the past year, diabetes, smoking, physical exercise and reading frequency, sleep disorders, anxiety, depression, and social support were associated with the risk of MCI in older adults with hypertension (*P* < 0.05; Table 1).

Using the occurrence of MCI as the dependent variable (0 = no, 1 = yes), and using the 16 factors identified as statistically significant in the univariate analysis as the independent variables, binary logistic regression analysis (forward-logistic regression conditional method) was conducted. The results showed that grade 2 hypertension, diabetes, years of hypertension, history of stress, smoking, and sleep disorders were risk factors for MCI in older adults with hypertension. Education level, physical exercise, reading, social support, and better medication compliance were protective factors against MCI in these patients (*P* < 0.05), as shown in Table 2.

**Table 2:** Multivariate analysis of factors influencing mild cognitive impairment risks in older adults with hypertension

Characteristics	*β*	*SE*	$\mathbf{Ward}^{\mathbf X^{\mathbf2}}$	*P*	*OR*	95% CI
Grade 2 hypertension	0.993	0.347	8.205	0.004	2.699	1.368 ~ 5.324
Technical Secondary school/High school	-2.595	0.682	14.472	< 0.001	0.075	0.02 ~ 0.284
College or above	-3.118	0.741	17.704	< 0.001	0.044	0.01 ~ 0.189
Diabetes	1.464	0.375	15.227	< 0.001	4.321	2.072 ~ 9.012
Hypertension Course	1.264	0.227	30.991	< 0.001	3.539	2.268 ~ 5.522
Have a history of stress in the past year	1.549	0.409	14.32	< 0.001	4.709	2.111 ~ 10.507
Smoking	0.994	0.338	8.659	0.003	2.703	1.394 ~ 5.243
Regular physical exercise	-1.965	0.504	15.206	< 0.001	0.14	0.052 ~ 0.376
Reading (Occasionally)	-0.895	0.452	3.915	0.048	0.409	0.168 ~ 0.992
Reading (Often)	-1.756	0.88	3.982	0.046	0.173	0.031 ~ 0.969
Social support	-0.063	0.03	4.469	0.035	0.939	0.886 ~ 0.995
Sleep disorder	0.826	0.361	5.217	0.022	2.283	1.124 ~ 4.637
High medication compliance	-0.828	0.274	9.103	0.003	0.437	0.255 ~ 0.748

Cox Snell R2 = 0.462, Nagorno R2 = 0.620, F = 65.47, *P* < 0.001

*Β* regression coefficient, *SE* standard error, *CI* confidence interval, *OR* odds ratios

### Development of a nomogram model

**Fig. 1:**
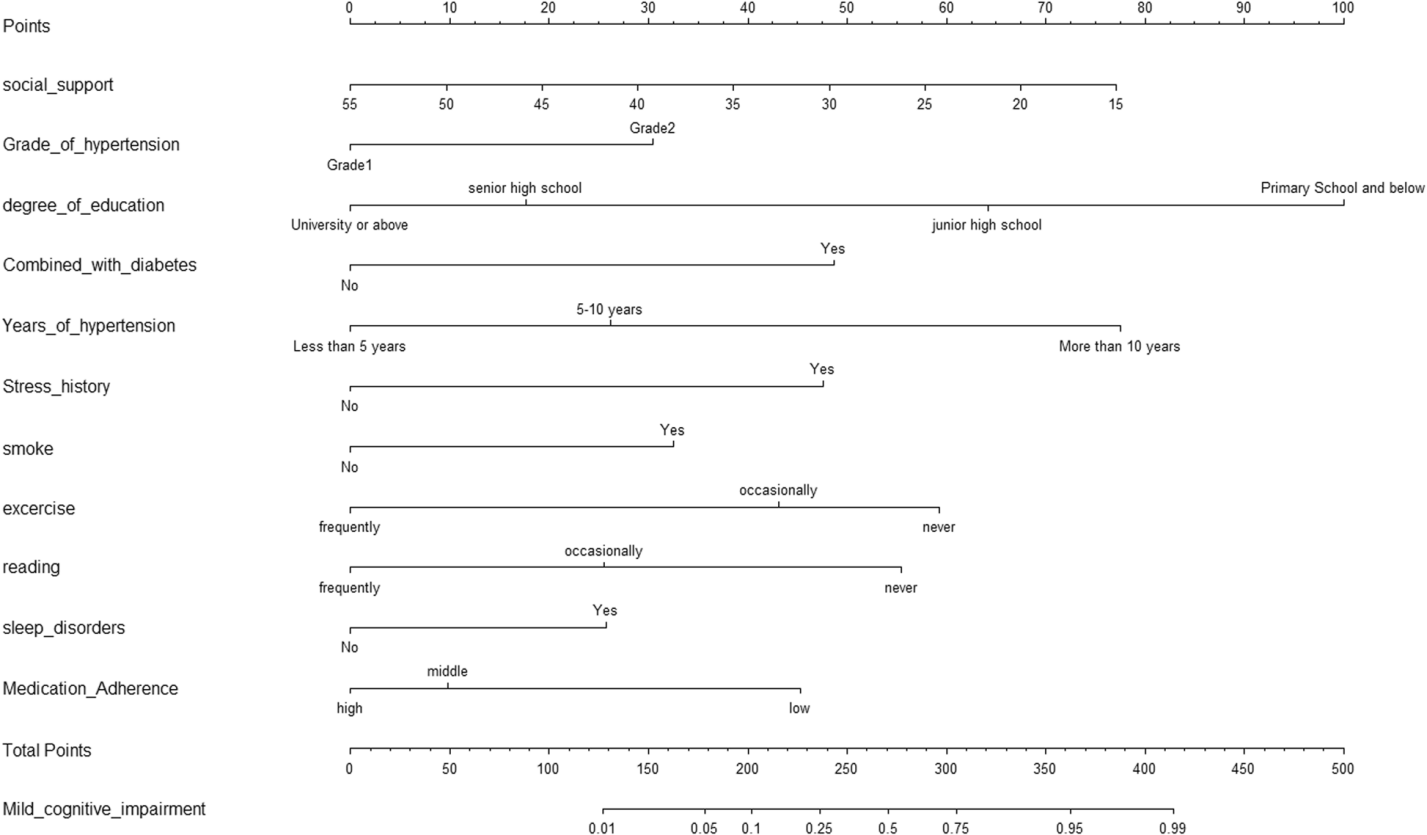
Nomogram predicting the MCI of older adults with hypertension

A nomogram was drawn according to the influencing factors determined by the above multivariable analysis (Fig. 1). A vertical line was drawn at the top of the nomogram according to different variables to obtain the corresponding score; the scores of each variable were added to obtain the total score, and then the corresponding total risk score was obtained at the bottom end of the nomogram. For example, if an older adult with hypertension had a social support score of 55, a score of 0 was assigned. Grade 2 hypertension was assigned a score of 30. A Bachelor’s degree was assigned a score of 0. The presence of diabetes was assigned a score of 50. If the duration of hypertension was < 5 years, a score of 0 was assigned. If there was no stress history, 0 points were scored. Smoking added 32.5 points to the score. If the patient exercised occasionally, they scored 45 points; if they occasionally read, they scored 25 points. The absence of a sleep disorder resulted in no additional points added to the total score. If medication compliance was average, 10 points were added. For the patient described above, the total score would be 192.5, and the probability of MCI corresponding to this total score on the nomogram (Fig. 1) would be between 0.05 and 0.1. The probability of MCI in older adults with hypertension was predicted to be between 5 and 10%.

### Verification of the nomogram model

**Fig. 2:**
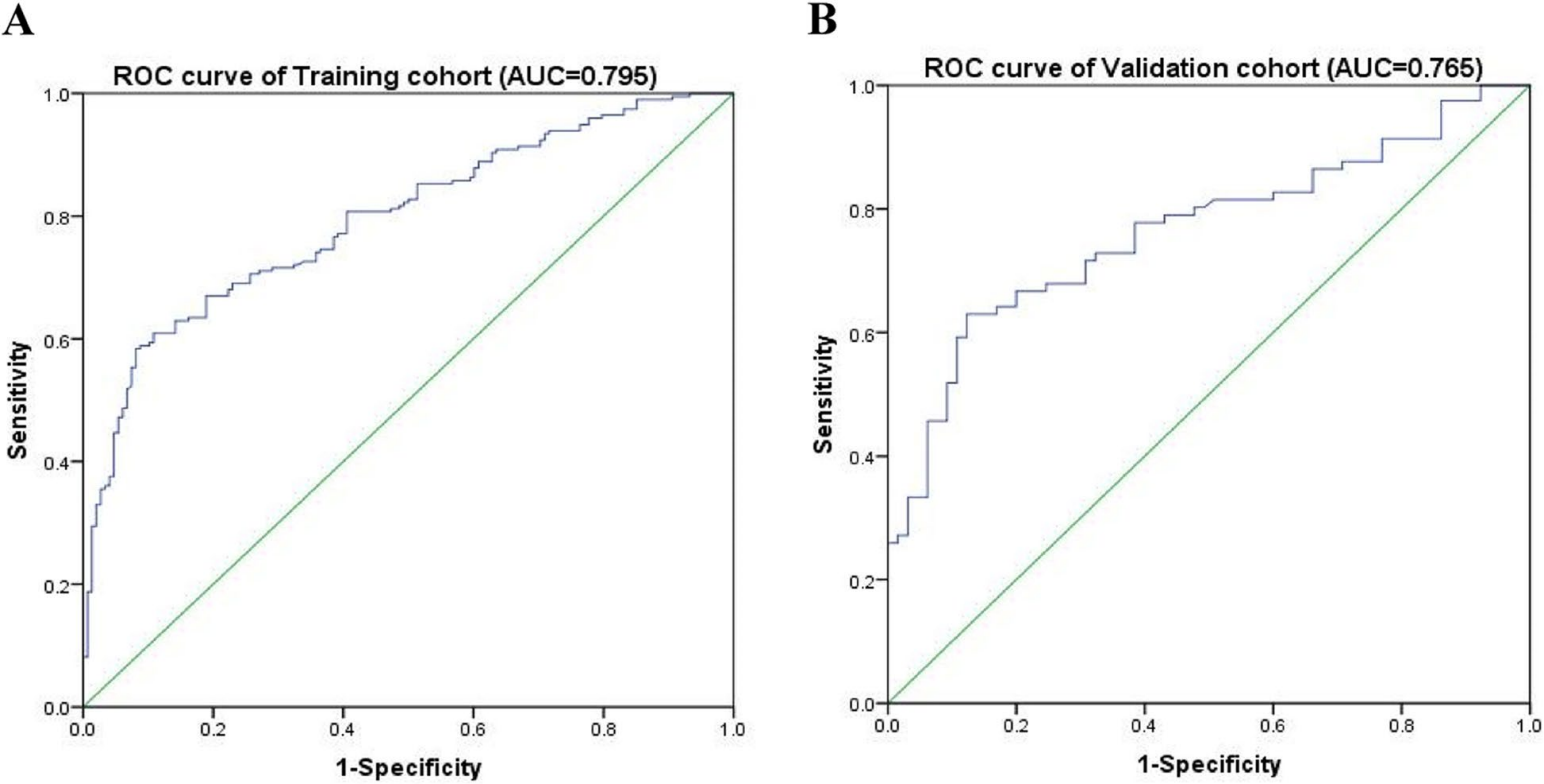
The ROC curve of the nomogram for predicting MCI in the training cohort (**A**) and the validation cohort (**B**). Note: AUC: the area under the ROC curve; MCI: mild cognitive impairment; ROC: receiver operating characteristic

**Fig. 3:**
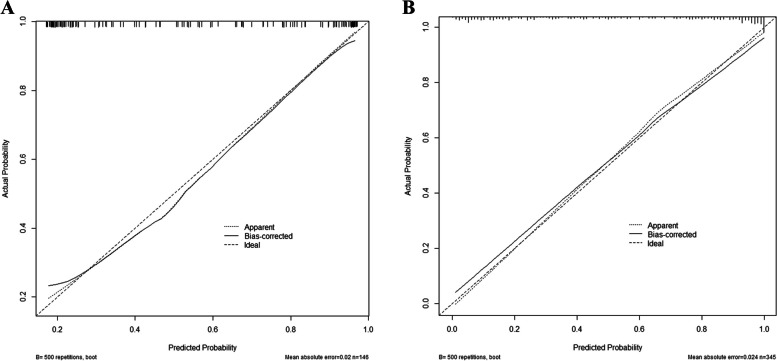
Calibration plot for predicting MCI in the training cohort (**A**) and the validation cohort (**B**)

The ability of the model to discriminate MCI in older adults with hypertension was then evaluated by the area under the ROC curve (AUC). The results showed that the AUC of the modeling group was 0.795 (95% confidence interval [CI]: 0.749–0.841), which showed that the model’s predictive ability is good. The Jordan index of the ROC curve was 0.503, sensitivity 0.584, specificity 0.919, positive-predictive value 90.53%, and negative-predictive value 62.50% (Fig. 2A). In the validation group, the area under the ROC curve was 0.765 (95% CI: 0.688–0.842), Jordan index was 0.507, sensitivity was 0.63, specificity was 0.877, positive-predictive value was 84.46%, and negative-predictive value was 65.52%, indicating that the model had good accuracy (Fig. 2B). The validity of the prediction model was evaluated by curve fitting. Good fitting was obtained in both groups (modeling group: *P* = 0.98; validation group: *P* = 0.96). In the modeling validation groups, the difference between the model fitting curve and the ideal curve data was not statistically significant, indicating that the prediction probability of the model was consistent with the actual probability and had good accuracy, as shown in Fig. 3.

## Discussion

### Risk factors related to the risk of MCI in older adults with hypertension

This study showed that higher education (Technical Secondary school/High school/College or above) was a protective factor against MCI in older adults with hypertension. These results are similar to those of Yating et al. [21]. This finding may be related to the plasticity of the central nervous system in the human brain. Through education, cognitive functions, such as abstract thinking and logical reasoning, can be developed to a certain extent, protecting neurons and improving cognitive functions.

At present, there is no unified research on whether the cognitive function of patients with hypertension will gradually decline with age. Some cross-sectional studies and longitudinal studies show that hypertension in old age has a significant adverse effect on overall cognition and specific cognitive areas such as executive ability, but some studies also show that the attention function and memory function of middle-aged hypertensive group are more obvious than that of elderly hypertensive group [22]. The univariate results of this study show that the difference between age and cognitive function is statistically significant, but the multivariate results show that it is not the influencing factor of cognitive function in older patients with hypertension, which may be related to the small sample size and the limited age of the subjects in this study. The specific relationship between the two needs further study and discussion. Therefore, it is suggested that medical staff should pay attention to the management of cognitive function of hypertensive patients with old age and low educational level, identify whether MCI occurs as soon as possible, intervene in advance and delay the development of the disease.

This study showed that secondary hypertension, a longer duration of hypertension, and diabetes mellitus were risk factors for MCI development in older adults with hypertension. This was consistent with the results of Anto et al. [23]. By investigating the cognitive status of older patients with different grades of hypertension, the results showed that the cognitive levels of these patients were significantly related to the classification and severity of hypertension. Regarding the course of hypertension, Li et al. [4] showed that the incidence and severity of cognitive dysfunction in patients with hypertension correlated positively with the course of hypertension: the longer they had hypertension, the less their cognitive abilities. The reason may be related to hypertension leading to arteriosclerosis, blood pressure fluctuation, decreased blood flow and white matter damage [24, 25], etc. Too high blood pressure can lead to changes in cerebrovascular structure, vascular dysfunction and the destruction of blood–brain barrier integrity to a certain extent, thus reducing cognitive function. Moreover, studies have demonstrated that diabetes is an independent risk factor for MCI, with a complex mechanism, which may be related to impaired insulin signal transduction, inflammation, accumulation of advanced glycation end-products, and oxidative stress [26].

Hypertension is a lifelong disease; once the patient becomes ill, it must be treated over the long term. The results of this study showed that good compliance with hypertension drugs in older adults with hypertension protects against MCI, which is consistent with previous research results [27]. Good medication compliance implies that the patients take their medications regularly, controlling blood pressure at a relatively stable level and reducing damage to cognitive function to a certain extent. Therefore, it is suggested that medical staff should strengthen health education on drug therapy for older adults with hypertension, emphasize the importance of drugs, improve their attention to drugs, and then control blood pressure and reduce the damage of cognitive function.

Regular physical exercise or reading was also a protective factor for cognitive function, consistent with the results of Wei et al.’s results [16]. Exercise habits also have an impact on cognitive function. This may be because physical exercise can improve nerve plasticity and weaken nerve degeneration. Simultaneously, physical exercise can enhance nerve function, including neurotransmitter and neurotrophin functions, cause changes in the cerebrovascular system, provide nerve protection, and reduce nerve degeneration.

Social support was another protective factor against MCI in older adults with hypertension, similar to the results of Yun and others [28]. This may be related to the cognitive reserve mechanism. Social support can promote the formation of synapses in the brain by providing cognitive or mental stimulation, thus playing a compensatory role in maintaining normal cognitive function [29]. At the same time, it is mentioned in the buffer effect model of social support that when individuals are under high-intensity pressure, social support can buffer the damage of stress events to their physical and mental health in time, and can play an active role in helping individuals promote their physical and mental health, thus reducing the damage of their cognitive function to some extent.

Studies have shown that nicotine can temporarily improve the brain's attention, reaction speed, and short-term memory [30], which may be related to the role of acetylcholine receptors; however, long-term smoking can cause an obvious decline in cognitive function and even dementia, which may be because smoking can reduce the areas of gray matter and white matter in the brain, thus affecting cognitive function.The results of this study showed that smoking is a risk factor for MCI in older patients with hypertension. Besides active smoking, long-term exposure to second-hand smoke will also cause cognitive impairment. It is suggested that smoking will decrease the cognitive function of elderly hypertensive patients in community.

This study further showed that sleep disorders are a risk factor for MCI in older adults with hypertension. Shorter sleep time causes cognitive function to decline faster, to some extent, and neurodegenerative diseases may also occur, which increases the risk of dementia [31]. The dentate gyrus of patients with insomnia shrinks due to a decrease in the number of neurons, which further affects cognitive function.

Therefore, clinical medical staff should encourage patients to establish healthy living habits. Quitting smoking and ensuring high-quality sleep can improve cognitive function in older patients with hypertension.

### Predictive ability of the nomogram model

In this study, 11 factors were selected through logistic regression analysis to build a prediction model. The nomogram was based on the score of 11 risk factors, and the total score was obtained by adding the scores of the 11 risk factors. The predicted probability of MCI in older adults with hypertension was easily obtained based on the total score. Simultaneously, this study evaluated the model's AUC, sensitivity, specificity, positive-predictive value, and negative predictive value by ROC curve by evaluating people from different communities for internal and external validation. The results indicated that the overall predictive ability of the model was good. In the era of precision medicine, where individualization is paid more and more attention, nomogram can integrate multi-source clinical information, provide accurate and visual risk prediction, assist clinicians to formulate targeted individualized treatment plans, and help patients' families to understand the prognosis and improve compliance.

### Limitations

A limitation of this study is that it was a cross-sectional study, and the data were collected simultaneously. The sequence and causal relationships between various factors may have caused deviations in the results. Therefore, clinical or basic studies should be conducted to verify the conclusions. In addition, because of the limited research time, the sample size of this study is relatively small. A multi-center and large-sample study will be conducted to further explore the relationship between hypertension and MCI.

## Conclusions

This study constructed a nomogram model for predicting MCI risk in older adults with hypertension. After internal and external verification, the model exhibited good prediction efficiency. It provides a simple and intuitive scoring table for clinical medical staff that allows rapid and accurate assessment of the risk of patients with hypertension for MCI development. This can improve their awareness of early warnings of MCI, prevent and delay the occurrence and development of MCI, avoid or slow down the occurrence of dementia, and improve the quality of life of older adults with hypertension.

## Data Availability

The datasets used and/or analysed during the current study available from the corresponding author on reasonable request.

## Abbreviations

ADL: Activities of Daily Living
AUC: The area under the receiver operating characteristic curve
CI: Confidence interval
MCI: Mild cognitive impairment
MMSE: Mini-Mental State Examination
MoCA: Montreal Cognitive Assessment
OR: Odds ratios
ROC: Receiver operating characteristic
